# 146. Predictors of Long Duration Antibiotic Therapy for Urinary Tract Infections and Community-Acquired Pneumonia in Pediatric Ambulatory Care Settings

**DOI:** 10.1093/ofid/ofab466.348

**Published:** 2021-12-04

**Authors:** Rohan M Shah, Shan Sun, Tonya Scardina, Sameer Patel

**Affiliations:** 1 Northwestern University, West Chicago, IL; 2 Ann & Robert H. Lurie Children’s Hospital of Chicago, Chicago, IL; 3 Ann & Robert H. Lurie Children’s Hospital of Chicago/Northwestern University Feinberg School of Medicine, Chicago, IL

## Abstract

**Background:**

Significant variation exists in the duration of antibiotic therapy for children in ambulatory care settings. Understanding drivers of variation for common conditions such as community-acquired pneumonia (CAP) and urinary tract infection (UTI) is important to informing antimicrobial stewardship interventions.

**Methods:**

A retrospective observational study was conducted of patients with CAP and UTI seen in outpatient clinics or discharged from the emergency room (ER) of a tertiary care children’s hospital network from 2016 – 2019. Diagnoses CAP and UTI were identified via ICD-10 coding. Only oral medications ordered for ≥ 3 and < 28 days were included. Multivariable logistic regression was performed to identify predictors of long antibiotic duration (defined as ≥ 10 days). Potential non-clinical drivers of longer duration included race, ethnicity, sex, primary language, and insurance status.

**Results:**

A total of 2,104 prescriptions for CAP from 442 prescribers and 1,070 prescriptions for UTI from 314 prescribers were included. Antibiotic durations were ≥ 10 days in 59.9% and 47.6% of prescriptions for CAP and UTI, respectively. Long duration of therapy was more common in children discharged from the ER when compared to clinics for both CAP (OR 1.795, 95% CI: 1.107 - 2.929), and UTI (OR 5.149, 95% CI: 1.933 - 16.373). The proportion of patients with long duration of therapy increased with younger age for both diagnoses and decreased overall in the final year of the study. Race, gender, ethnicity, and primary language were not associated with prolonged duration of therapy. However, patients with Medicaid insurance were more likely to receive long duration of therapy for CAP (OR 1.337, 95% CI: 1.062 - 1.682) and UTI (1.654, 95%, CI: 1.181 - 2.325).

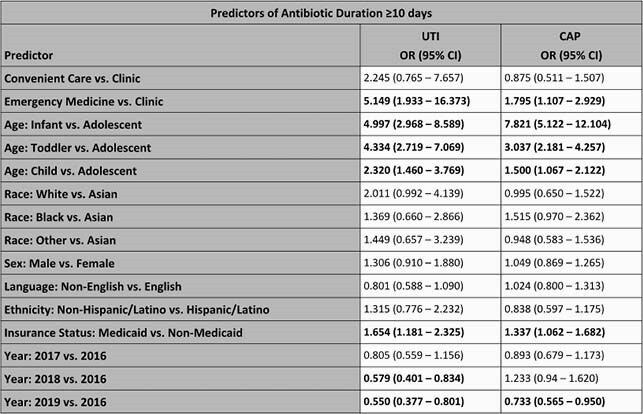

**Conclusion:**

In pediatric patients in ambulatory care settings, younger age, care in the ER, and being insured through Medicaid were independently associated with prolonged duration of therapy for both UTI and CAP.

**Disclosures:**

**All Authors**: No reported disclosures

